# Zika Virus: Origins, Pathological Action, and Treatment Strategies

**DOI:** 10.3389/fmicb.2018.03252

**Published:** 2019-01-07

**Authors:** Kirill Gorshkov, Sergey A. Shiryaev, Sophie Fertel, Yi-Wen Lin, Chun-Teng Huang, Antonella Pinto, Chen Farhy, Alex Y. Strongin, Wei Zheng, Alexey V. Terskikh

**Affiliations:** ^1^National Center for Advancing Translational Sciences, National Institutes of Health, Bethesda, MD, United States; ^2^Sanford Burnham Prebys Medical Discovery Institute, La Jolla, CA, United States

**Keywords:** ZIKV, re-purposing, *in vivo*, drugs, maternal transmission

## Abstract

The Zika virus (ZIKV) global epidemic prompted the World Health Organization to declare it a 2016 Public Health Emergency of International Concern. The overwhelming experience over the past several years teaches us that ZIKV and the associated neurological complications represent a long-term world-wide challenge to public health. Although the number of ZIKV cases in the Western Hemisphere has dropped since 2016, the need for basic research and anti-ZIKV drug development remains strong. Re-emerging viruses like ZIKV are an ever-present threat in the 21st century where fast transcontinental travel lends itself to viral epidemics. Here, we first present the origin story for ZIKV and review the rapid progress researchers have made toward understanding of the ZIKV pathology and in the design, re-purposing, and testing–particularly *in vivo*–drug candidates for ZIKV prophylaxis and therapy ZIKV. Quite remarkably, a short, but intensive, drug-repurposing effort has already resulted in several readily available FDA-approved drugs that are capable of effectively combating the virus in infected adult mouse models and, most importantly, in both preventing maternal-fetal transmission and severe microcephaly in newborns in pregnant mouse models.

## Virus Origin, Epidemiology, and Areas of Dissemination

Mosquito-transmitted Zika virus (ZIKV) is a member of the Flavivirus genus in the *Flaviviridae* family. Similar to other flaviviruses, ZIKV is transmitted by several mosquito species including *Aedes africanus, Ae. aegypti, Ae. albopictus*, and *Ae. hensilli* (Dick et al., 1952; Marchette et al., 1969; Grard et al., 2014; Ledermann et al., 2014).

ZIKV was first isolated in a sentinel rhesus monkey in the Zika forest of Uganda in 1947 (Dick et al., 1952). Prior to its rapid spread across the Pacific Islands in the 21st century, ZIKV was also documented in a handful of individuals in sub-Saharan Africa and then in Southeast Asia by the mid-20th century (Gatherer and Kohl, 2016). These cases were rare, geographically isolated events and many others may have gone undocumented. After its original identification, the first major ZIKV outbreak outside of Africa took place in 2007 on Yap Island with approximately three-fourths of the population over the age of three suspected to be infected. *Ae. hensilli* was the predominant mosquito species identified in the transmission of ZIKV in this event and infection was characterized by rash, arthralgia, and conjunctivitis (Duffy et al., 2009).

In October 2013, a larger ZIKV outbreak (29,000 cases) occurred in French Polynesia. While most patients exhibited mild symptoms, one patient developed Guillain-Barre Syndrome (GBS) 1 week post-infection. GBS is an auto-immune disease that attacks the peripheral nervous system leading to weakness in the extremities and, in severe cases, paralysis and death. Alarmingly enough, the total number of the GBS cases that followed this outbreak was roughly 20-fold higher than baseline levels, suggesting a direct association between ZIKV and GBS. Following this event, outbreaks were reported in New Caledonia, the Cook Islands, and the Easter Island (Musso et al., 2014a). Phylogenetic analyses of ZIKV variants isolated from French Polynesia (2013), Cambodia (2010), and Yap Island (2007) confirmed that these outbreaks were linked to the expansion of the Asian lineage of ZIKV (Cao-Lormeau et al., 2014).

The beginning of a string of severe outbreaks in the Americas ensued in March 2015 when ZIKV was detected in Bahia, Brazil (Campos et al., 2015). Between January and July 2015, the state of Rio de Janeiro also experienced its first ZIKV outbreak with patients who presented with headache, arthralgia, myalgia, non-purulent conjunctivitis, and lower back pain (Brasil et al., 2016). After reaching Brazil, the virus then rapidly spread throughout the Americas. Puerto Rico experienced its first case on December 31, 2015, and since then has experienced a total of 37,000 locally transmitted symptomatic or probable cases. The World Health Organization declared ZIKV a Public Health Emergency of International Concern on February 1, 2016 (Heymann et al., 2016). By February 2016, the CDC received nine confirmed ZIKV cases in pregnant women who had traveled to South America (Meaney-Delman et al., 2016). By April of 2016, ZIKV transmission had been registered in 27 countries in the Americas (Paixão et al., 2016). The first locally transmitted ZIKV case in the United States mainland was reported in July of 2016 (Ventura et al., 2016). By July 22, 2016, the Florida Department of Health had identified 321 ZIKV disease cases in Florida residents and visitors (Likos et al., 2016). Currently, the CDC reports there have been 5,716 cases reported on the United States mainland, with 231 of those cases transmitted locally through mosquitos (CDC Cumulative Zika Disease Case Counts in the United States, 2015–2018).

There are currently 86 countries, territories, or subnational areas with evidence of ZIKV transmission (http://www.who.int/emergencies/zika-virus/classification-tables/en/). As ZIKV advances across the globe, more imported cases of human infection are being reported worldwide in places with no previous record. For example, in 2016 the first Korean case of imported ZIKV infection was identified in a 43-year-old man (Jang et al., 2016). Spain reported its first imported case in January of 2016 (Bachiller-Luque et al., 2016). Nine imported ZIKV cases were also detected in mainland China from February 1st to the 29th of 2016 (Dai et al., 2016). New Zealand reported a special case that involved a 47-year-old man who returned to the country with acute ZIKV infection and a concurrent onset of GBS (Siu et al., 2016). In 2017, the United States, including US territories, saw 1,080 cases with six occurring through sexual contact (Centers for Disease Control, 2017). According to the CDC, from January 1 to August 1, 2018 there have been a cumulative 108 ZIKV cases in the US with 74 of those occurring through local mosquito-borne transmission in Puerto Rico (Centers for Disease Control Prevention, 2018). Cumulatively, the harsh reality of ZIKV is evident. As of January 4, 2018, there have been 223,477 confirmed cases of ZIKV worldwide (Organization PAH, 2018). The actual number of infections is certainly higher as approximately 80% of infections are asymptomatic and the community lacked suitable diagnostic methods during the early outbreaks. The recurrence of ZIKV around the world may have several causes including the generation of more virulent strains, new routes of transmission, and novel modifiers of the disease (Koppolu and Shantha Raju, 2018).

For further information on ZIKV history and epidemiology, we refer readers to excellent review articles recently published within the last 2 years (Weaver et al., 2016; Aliota et al., 2017).

## Virus Transmission, Clinical Symptoms, and Routes of Infection

ZIKV infection frequently goes unnoticed or is asymptomatic in approximately 80% of cases and most patients present with only mild symptoms (Hamel et al., 2015; Petersen et al., 2016). ZIKV is most commonly transmitted from the bite of a mosquito, where initial infection most likely occurs in human skin cells directly affecting permissive human dermal fibroblasts, epidermal keratinocytes, and immature dendritic cells (Hamel et al., 2015; Olagnier et al., 2016; Kim et al., 2018).

Experimental evidence suggests that in mice, ZIKV infects placental cells via a trans-placental route that results in restricted intrauterine growth (Miner et al., 2016; Weisblum et al., 2017). This route has been suggested to involve viral spread to the chorionic villi, amniochorionic membranes, and from the basal to the parietal decidua (Tabata et al., 2016). In 2016 it was estimated that 29% of babies born to infected mothers exhibit developmental abnormalities, suggesting that the trans-placental route of transmission, also termed vertical transmission in mice, is relevant to ZIKV infection in humans (Brasil et al., 2016). A very recent CDC evaluation of past cases in newborns states that one in seven is affected by vertical transmission (CDC Vital Signs report, August 8, 2018). Placental ZIKV infection induces proliferation and prominent hyperplasia of Hofbauer cells in the chorionic villi, but does not elicit villous necrosis or a lymphoplasmacellular or acute inflammatory reaction in maternal or fetal cells (Rosenberg et al., 2016). These developmental abnormalities tend to occur more frequently when infection takes place during the first trimester (Dang et al., 2016). Within the past 2 years, however, two cases were described in women infected during the third trimester, with fetuses displaying the first signs of congenital brain malformations at the 36th week of pregnancy (Soares de Souza et al., 2016). While one study found that primary human trophoblasts from full-term placenta do not host ZIKV because of their interferon gamma 1 response (Bayer et al., 2016), a different study revealed that ZIKV infection increased the risk of latent microcephaly because of the virus' (strain FLR, Colombia, 2015; Lahon et al., 2016) ability to replicate in human placental trophoblasts without causing any observable host cell destruction (Aagaard et al., 2017). Therefore, the congenital transfer of the virus by transplacental infection may cause this spectra of abnormalities (Oliveira Melo et al., 2016). One clinical study found that 14% of ZIKV-infected symptomatic semen samples were positive for ZIKV RNA and persisting for more than six months (Mead et al., 2018). Two other non-vector routes of ZIKV transmission include perinatal and sexual transfer (Besnard et al., 2014; Musso et al., 2015). A mouse model revealed that the infectious virus was present in the semen at days 7 to 21 post-inoculation (Duggal et al., 2017). Another report found human semen with viral up to 38 days after initial detection in humans (Medina et al., 2018). Evidently, the vaginal tract in mice is also highly susceptible to ZIKV replication (Yockey et al., 2016). There is high probability that this is also true for humans (Musso et al., 2015; Maxian et al., 2017; Sherley and Ong, 2018). Blood transfusion represents another worrisome mode of transmission, as two cases of probable transmission have already been reported in Brazil (Barjas-Castro et al., 2016; Counotte et al., 2018).

Accentuating the problem, presentations of ZIKV are appearing in cells and tissues that have not been previously seen in humans. In Brazil, a study detected the infectious ZIKV particles in the urine and the saliva of patients during the acute phase of infection. In a couple returning from Martinique, French West Indies, ZIKV remained detectable in the plasma for roughly two weeks; their urine samples tested positive at 39 days (Fourcade et al., 2016; Paz-Bailey et al., 2017).

Furthermore, it has been reported that two patients with ZIKV infection also had cases of severe thrombocytopenia. The first patient exhibited only 1,000 platelets/mm^3^ and died following multiple hemorrhages. The second patient had 2,000 platelets/mm^3^, melena, and ecchymosis, but, luckily, recovered after receiving intravenous immunoglobulin (Sharp et al., 2016). Another case reported severe immune thrombocytopenic exacerbation and antinuclear antibody positivity induced by ZIKV infection (Zea-Vera and Parra, 2016). In addition to thrombocytopenia, ZIKV is likely associated with ocular abnormalities. A case study in Salvador, Bahia, Brazil, revealed that in 29 examined infants born to ZIKV-infected pregnancies, ocular abnormalities were present in 17 eyes of 10 children (de Paula Freitas et al., 2016). ZIKV was also detected in the conjunctival fluid (Sun et al., 2016) suggesting that ZIKV affects the permeability of the retinal pigment epithelium (Salinas et al., 2017). A recent clinical case also associated the virus with hearing loss. A newborn from Brazil, whose mother was infected with ZIKV during pregnancy, was confirmed to have bilateral profound hearing loss (Leal et al., 2016a). A later retrospective testing (November 2015–May 2016) of 0–10 month-old infants with microcephaly and ZIKV infection revealed that 5 out of 70 children had sensorineural hearing loss (Leal et al., 2016b).

As mentioned above, GBS is a severe complication in adult ZIKV infection. Previous research provides evidence that further supports a direct association of ZIKV with GBS (Musso et al., 2014b). In a case study from December 2015 to March 2016, 19 patients in Cucuta, Columbia, with a recent history of acute viral syndrome compatible with ZIKV were found to exhibit signs of GBS. The symptoms developed in roughly 10 days after the onset of viremia (Arias et al., 2016). An additional study (Puerto Rico, January 1–July 31) identified 56 suspected cases of GBS. 34 (61%) of these patients had evidence of ZIKV or flavivirus infection (Dirlikov et al., 2016). Accordingly, there is a suspicion that anti-ZIKV antibodies cross-react with certain CNS/PNS proteins in infected adults and that these interactions contribute to the ZIKV-associated onset of neuropathological symptoms (Lucchese and Kanduc, 2016). Another contributing factor may be the long-term presence of ZIKV in the cerebrospinal fluid that persists for weeks after the clearance of peripheral ZIKV (Aid et al., 2017). GBS predominantly occurs during the post-infectious period with a delayed onset. However, this is not always the case, as there is a patient in whom an acute sensory polyneuropathy occurred during the active infectious phase (Cao-Lormeau et al., 2016; Medina et al., 2016).

Normally, ZIKV infection is not fatal. However, the first fatal case of ZIKV-associated encephalitis was reported in 2016 in a 47 year old non-pregnant woman (Soares et al., 2016) soon followed by the report of three additional ZIKV-related fatalities with one of the patients being severely immunocompromised (Zonneveld et al., 2016).

**Figure 1:**
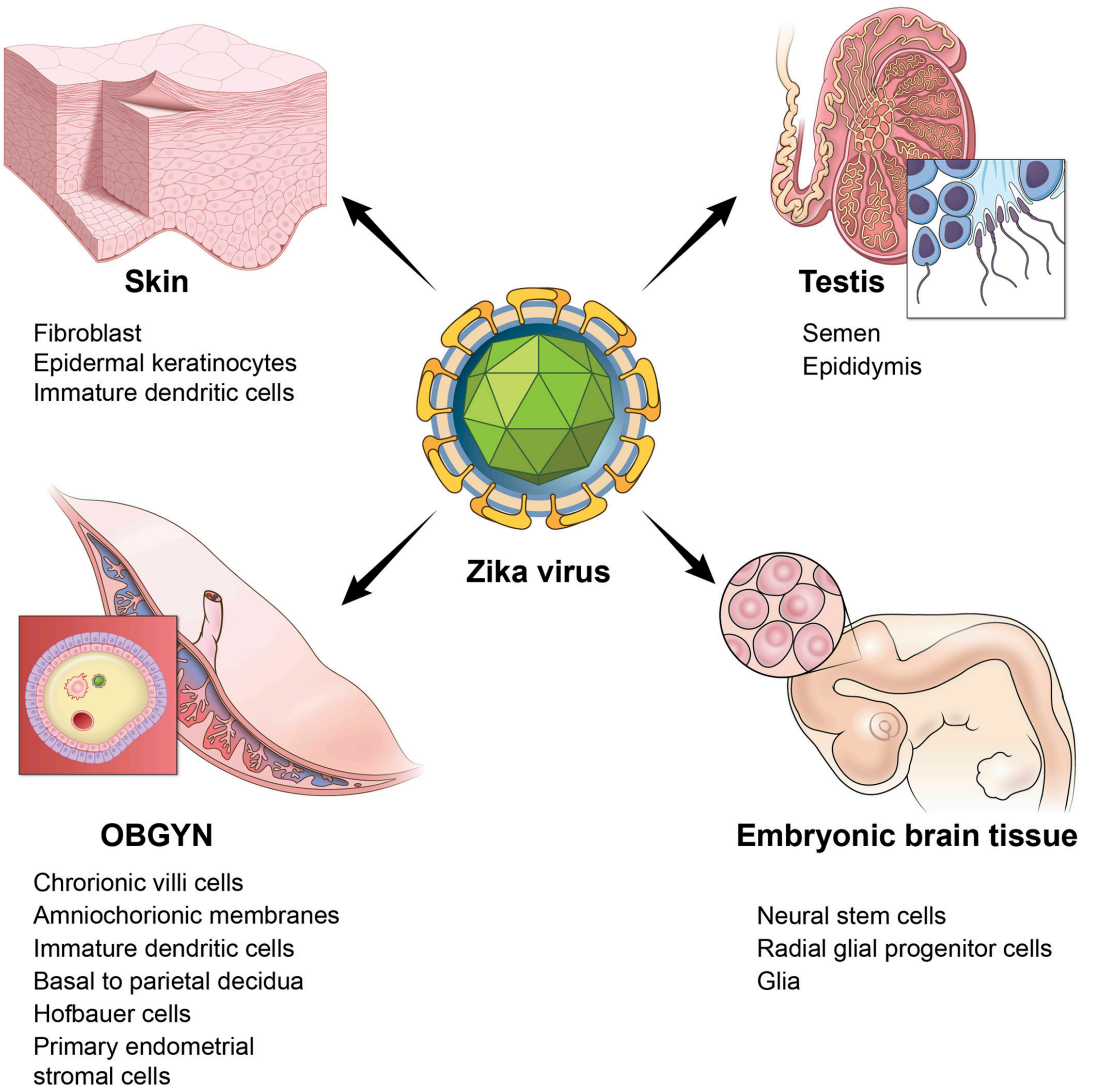
ZIKV infects multiple tissues in humans. Illustration depicting the tissue and cell types that are targeted by ZIKV. Skin is represented by a cartoon of a fibroblast cell. OBGYN tissues are represented by a cartoon of the villus of the placenta and its different cell layers, embryonic brain tissues depicted by a cartoon of the embryonic brain with neural progenitor cells, testis are depicted by a cartoon drawing of spermatozoa.

An additional risk of ZIKV in adults is damage to the testis. A study in mice reported the persistence of ZIKV in the testis and epididymis leading to extensive tissue damage. Male mice were reported to exhibit oligospermia, diminished testosterone, and inhibin B levels (Govero et al., 2016). A more recent study reinforced these observations and revealed that peritubular spermatogonium cells are vulnerable to ZIKV infection (Ma et al., 2017). Furthermore, even an acute, uncomplicated, symptomatic ZIKV infection may result in microhematospermia in the absence of hematuria (Torres et al., 2016). In women, primary human endometrial stromal cells are also infected by ZIKV (Pagani et al., 2017). Figure 1 depicts the multiple sites of ZIKV infection and some of the relevant cell and tissue types in humans.

In addition to widely studied congenital infection with ZIKV, recently studies have documented ZIKV infection in immunocompetent neonates (Li et al., 2018). Curiously, BALB/c neonatal mice were resistant to ZIKV infection, whereas Kunming, ICR and C57BL/6 neonatal mice were fatally susceptible to ZIKV infection (Li et al., 2018). Similar to congenital infection, different areas of CNS including gray matter, hippocampus, cerebral cortex, and spinal cord (but not olfactory bulb) were severely affected. ZIKV was replicated and caused pathogenesis in liver and testis, implying that ZIKV infection may engender a high health risk in neonates by postnatal infection (Li et al., 2018).

## Molecular and Cellular Mechanisms of ZIKV Pathogenesis

Evidence for a ZIKV-induced dysregulation of neural development comes from the studies in which ZIKV was injected directly into the lateral ventricle of the fetal mouse brain. The virus readily homed to the dorsal telencephalon while other brain regions were spared. There was also inhibition of cell division in the ventricular, sub-ventricular and intermediate zones; the sub-ventricular zone is a home to many radial glial progenitor cells that differentiate into cortical neurons (Petreanu and Alvarez-Buylla, 2002). The infected cortex became shorter and the size of the lateral ventricle cavities was noticeably reduced as compared with uninfected mice (Nguyen et al., 2016). In addition, after entering the CNS, ZIKV infects astrocytes, visual system nuclei, and the retina (van den Pol et al., 2017).

Undifferentiated neurons, which are plentiful in the fetal brain, are highly susceptible to ZIKV infection, especially during the early stages of neurogenesis. Intriguingly, only 40% of human fetal neural progenitors become infected by ZIKV, thus limiting the anti-ZIKV immune and inflammatory response (Hanners et al., 2016; Rolfe et al., 2016). However, ZIKV infection readily leads to the death of these infected cells (Souza et al., 2016).

In contrast, differentiated neurons in the adult brain are relatively resistant to the virus. These observations explain the infrequent neurological pathologies in the infected adults (Hughes et al., 2016). However, in a 6-week-old Irf3^−/−^ Irf5^−/−^ Irf7^−/−^ triple knockout mouse model, the adult NCSs in the sub-ventricular zone of the anterior forebrain and the sub-granular zone of the hippocampus became infected with ZIKV, suggesting that certain neural cells in adults are susceptible to ZIKV pathology (Li et al., 2016b). The inconsistent results between different research groups indicates this aspect of ZIKV infectivity is not completely understood and warrants further study, particularly for the blood-brain barrier permeability to viruses. Differences in blood-brain barrier permeability in different stages of fetal development may help to explain the greater susceptibility of fetuses to ZIKV in the earlier trimesters.

There is overwhelming experimental support for a direct link between ZIKV infection and microcephaly in both rodent models and humans. Thus, Asian ZIKV (Asian strain SZ01) infects neural progenitor cells (NPCs) *in vivo* and affects brain development resulting in cell-cycle arrest, cell apoptosis, differentiation inhibition, cortical thinning, and, ultimately, microcephaly. Further evidence comes from the genome-wide profiling analyses of the infected brain suggesting the up-regulation of the viral entry receptor genes and aberrations in the immune response, apoptosis, and microcephaly pathways (Kumar et al., 2016; Li et al., 2016b).

Naturally, ZIKV shares some common biology with other flaviviruses such as West Nile (WNV), Dengue (DENV), tick-borne encephalitis, and Yellow fever, which may also cause neurological damage and encephalitis (Solomon, 2004). However, the striking and unusually dangerous epidemiology of ZIKV and its unique interactions with humans suggest the presence of a modified underlying molecular makeup of this formidable virus. It has now been firmly established that there is a causal relationship between prenatal ZIKV infection and microcephaly, along with other extensive neurological and brain anomalies (Li et al., 2016a, 2017b; Melo et al., 2016; Nishiura et al., 2016). It is likely that the Toll-like receptor 3 (TLR3) is linked to ZIKV neuropathology as TLR3 was upregulated in human ZIKV-infected organoids and mouse neurospheres. Conversely, TLR3 inhibition reduced the phenotypic effects of ZIKV infection. The pathway analysis suggested that ZIKV-related TLR3 activation affected 41 neurodevelopmental genes (de Araújo et al., 2016; Rasmussen et al., 2016). Based on this and similar studies, there is now a consensus among researchers that ZIKV predominantly targets neural progenitors and affects neurogenesis during the first trimester and, as a result, abrogates normal brain development (Dang et al., 2016; Tang et al., 2016). In agreement, immunocytochemistry and electron microscopy demonstrated that ZIKV infects neurospheres and brain organoids, reducing their viability and growth (Garcez et al., 2016). In order to mimic ZIKV infection during the course of human cortical development, researchers employed organotypic cultures and demonstrated that ZIKV preferentially infected neural progenitors in early stage cortical organoids (Xu et al., 2016a). Using cost-effective miniature spinning bioreactors, the authors demonstrated that ZIKV infection resulted in suppressed proliferation and increased cell death in the early stage cortical organoids. Not surprisingly, these organoids exhibited certain macroscopic features resembling microcephaly (Xu et al., 2016a).

Consistent with these findings, Wu et al. determined that ZIKV injected intraperitoneally into pregnant mice infected radial glial progenitor cells (RPCs) of the dorsal ventricular zone in their fetuses. RPCs are responsible for the development of the cortex and, in a result, their infection markedly reduced the number of fetal cortical founder cells. Infected fetal mice exhibited a reduced lateral ventricle cavity volume and a discernable decrease in cortical surface area. Overall, there is a well-justified conclusion that ZIKV selectively affects the fetal brain development (Brault et al., 2016; Wu et al., 2016).

Nowakowski et al. (2016) examined the expression of receptors implicated in the ZIKV cell entry. They determined that the candidate viral entry receptor Axl is highly expressed by human RPCs, astrocytes, endothelial cells, and microglia in the developing human cortex, and by progenitor cells in the developing retina. It was also shown that Axl expression in radial glia is conserved in the developing mouse and ferret cortices, and in the human stem cell-derived cerebral organoids. In agreement, Axl was shown to mediate the productive infection of human endothelial cells (Liu et al., 2016). Curiously, a more recent study in Axl-null mice indicated that Axl may be just one of the several ZIKV receptors *in vivo* (Li et al., 2017c). Furthermore, a more recent work using knockout mice provided results that ZIKV showed no reliance on Tyro3, Axl, and MerTK (TAM) receptors for infection in mice (Hastings et al., 2017). Another recent study suggests Axl may promote ZIKV infection by antagonizing type I interferon signaling in primary human astrocytes rather than acting as a cell entry receptor (Chen et al., 2018). Over the last two years the controversy has grown over the influence of Axl on ZIKV infection and it remains a controversial topic in the field.

Additional host cell targets have recently been identified. Musashi 1, an RNA binding protein, was found to interact with ZIKV 3′ UTR stem loop structures in ZIKV PE243 (Asian-lineage) and MR766 (Ugandan-lineage; Chavali et al., 2017). The genome-wide CRISPR/Cas9-based screens suggest the endoplasmic reticulum-associated signal peptidase complex (SPCS) also is a pharmacological target for inhibiting ZIKV infection. Because ZIKV employs the host cell signal peptidase for its polyprotein processing, SPCS1 knockout greatly reduced the yield of infectious ZIKV (Zhang et al., 2016b). The traditional anti-flaviviral drug targets such as NS2B-NS3 viral protease and NS5 RNA polymerase are also considered to be promising targets for ZIKV inactivation (Wang et al., 2017a).

Experimental evidence suggests that in mice, ZIKV^BR^ (Brazilian strain) crosses the placenta and causes microcephaly by infecting the cortical progenitor cells and causing dysregulated autophagy resulting in apoptosis. The coincidence of these events also severely impairs neurodevelopment (Cugola et al., 2016).

Furthermore, ZIKV inhibits the Akt-mTOR pathway in fetal neural stem cells (NSCs), leading to defective neurogenesis and the aberrant activation of autophagy (Liang et al., 2016). The analysis of human neuroepithelial-like stem (NES) cells, organotypic fetal brain slices, and ZIKV-infected microencephalic brain samples revealed that ZIKV affected both neocortical and spinal NES cells as well as their fetal homolog, RPCs, causing disrupted mitoses, supernumerary centrosomes, structural disorganization, and cell death (Onorati et al., 2016). It is also likely that ZIKV stimulates cytokines in the infected cranial neural crest cells. This cytokine storm contributes to the death and aberrant differentiation of neural progenitors, affecting the signaling cross-talk among the developing brain regions and destroying the normal brain and facial development programs (Bayless et al., 2016).

Intriguingly, ZIKV has evolved to contain a nucleotide composition and RNA modifications, such as methylation and the formation of G-quadruplexes that allow effective replication in mosquito and primates. The ZIKV genome produces non-coding subgenomic flavivirus RNAs (sfRNAs) due to stalling of host 5′-3′ ribonucleases on viral RNA structures in the 3′ untranslated region (UTR). These sfRNAs exert important proviral functions such as antagonizing the innate interferon response and RNA interference; see excellent recent review for further details (Goertz et al., 2017).

A global proteome- and gene-expression wide view of ZIKV infection may provide the field with potential biomarkers and indicators of pathogenesis unseen with existing studies (Mao et al., 2016; Zhang et al., 2016a; Garcez et al., 2017). Further, applying this approach to vertical transmission may provide insights into the critical proteins in the brain involved in the development of microcephaly. To this end, our groups have embarked on a mission to characterize the changes to the proteome in ZIKV-infected mothers and their pups (Gorshkov et al., in preparation). Pathway analysis of ZIKV-infected pups *in utero* can uncover the alterations in key subsystems. Proteomics can also reveal essential molecules like kinases, receptors, and transcription factors to target with ZIKV therapy. Indeed, the major goal of this work will be to identify biomarkers for ZIKV-induced microcephaly.

## Possible Treatments and Drug Development Strategies

A commonly used approach for antiviral drug development involves targeting specific vulnerable stages of the pathogen's life cycle in order to disrupt its propagation within cells, effectively protecting cells and their neighbors from viral spreading (da Silva et al., 2018). In drug discovery, structure-based guidance is essential for a better understanding of the unique ZIKV pathology and selective drug design. The crystal structures of ZIKV helicase-ATP-Mn^2+^ and ZIKV helicase-RNA involved in viral replication reveal that upon RNA binding, rotation of the motor domains causes significant conformational changes (Luo et al., 2008; Tian et al., 2016). A long intertwined loop forming a hydrophobic “spike,” which can contribute to cellular membrane association was observed in the wing domain of ZIKV nonstructural protein 1 (NS1), a major host-interaction molecule (Xu et al., 2016b). Comparative studies with West Nile and Dengue virus NS1 structures reveal conserved features, but diversified electrostatic characteristics on both inner and outer faces suggesting different mechanisms of flavivirus pathogenesis (Xu et al., 2016b). A crystal structure of ZIKV NS2B-NS3 protease also provides an important structural template for inhibitor design and explains the substrate cleavage preferences of the ZIKV protease in a more detail (Phoo et al., 2016).

Until now, the research has been focused largely on either re-purposing of the currently available drugs or on the development of the novel, predominantly antibody-based, therapeutics. Because ZIKV and DENV share common epitopes, human anti-DENV monoclonal antibodies, and immune serum partially neutralize ZIKV (Swanstrom et al., 2016). These data increase the probability of designing an epitope-focused cross-neutralizing vaccine or an inactivated virus vaccine (Barba-Spaeth et al., 2016; Robbiani et al., 2017). In 2017, an inactivated virus vaccine against ZIKV (African strain MR 766) provided full protection against the homotypic and heterotypic ZIKV strains *in vitro* (Sumathy et al., 2017). However, thus far only one investigational vaccine developed at the National Institute of Allergy and Infectious Disease has entered phase 1 clinical trials (Abbasi, 2016). The process of drug development takes years and, unfortunately, the development, optimization, clinical trials, and production of the final product(s) will take significant time to reach patients.

Because of the potential for antibody-dependent enhancement (ADE) of viral infection, a phenomenon which has been previously described to enhance secondary DENV infections in patients vaccinated against DENV (Katzelnick et al., 2017), a vaccine approach requires an extreme caution because it is possible that pre-existing immunity to DENV may negatively impact the protective immune response against ZIKV (Priyamvada et al., 2016). The data suggest that the convalescent plasma from DENV- and WNV- infected patients and certain anti-DENV neutralizing monoclonal antibodies enhanced, rather than neutralized, ZIKV infection both *in vitro*, and in mice (Charles and Christofferson, 2016; Dejnirattisai et al., 2016; Paul et al., 2016; Bardina et al., 2017).

On the other hand, repurposing of the existing drugs is progressing rapidly. Resulting from high-throughput screens (HTS) using FDA-approved drug libraries, several FDA-approved compounds inhibiting ZIKV propagation in cultures have emerged. The list includes the following drugs: the pan-caspase inhibitor emricasan, the antihelmintic drug niclosamide, cyclin-dependent kinases inhibitors (Barrows et al., 2016; Xu et al., 2016a), epigallocatechin catechol gallate (EGCG) (Carneiro et al., 2016), cavinafungin (Estoppey et al., 2017), sofosbuvir, and related inhibitors of NS5 RNA polymerase activity (Elfiky, 2016; Ferreira et al., 2017; Sacramento et al., 2017), an anti-malarial drug chloroquine (Shiryaev et al., 2017), temoporfin (a chlorin-based photosensitizer drug currently used in photodynamic therapy for squamous cell head and neck carcinoma), the anti-parasitic nitazoxanide (a broad-spectrum antiparasitic and antiviral drug; Ferreira et al., 2017), and the antiprotozoal drug emetine (Ferreira et al., 2017; Yang et al., 2018). The HTS approach has also led to the discovery of novel drug candidates such as 6-azauridine and finasteride as well-several pyrimidine synthesis inhibitors such as brequinar as potent anti-ZIKV inhibitors (Adcock et al., 2017). Because the field of antiviral research is growing rapidly, many compounds have been identified *in vitro*. However, not all these molecules will make it to clinical trials due to lack of *in vivo* efficacy. Therefore, the following sections will describe ZIKV inhibitors validated *in vivo* and are summarized in Table 1. ZIKV inhibitors validated *in vitro*, including some of the *in vivo* compounds, along with their IC50, ZIKV strain, cell line, and assays used are summarized in Table 2.

**Table 1:** Small-molecule inhibitors of ZIKV confirmed in mice.

**Inhibitor**	**Dose**	**Effects**	**Mice**	**Reference**
**INHIBITORS OF VIRAL RNA REPLICATION AND TRANSLATION**				
Sofosbuvir	20 mg/kg/day; 50 mg/kg/day	Reduced viremia, doubled the survival time, prevented neuromotor impairment and loss of memory, blocked vertical transmission	Swiss albino, SJL	Ferreira et al., 2017; Mesci et al., 2018
Temoporfin	1 mg/kg	Reduced viremia, protected against lethal challenge	BALB/C, A129	Sacramento et al., 2017
7-deaza-2′-C-methyladenosine	50 mg/kg/day	Delayed virus-induced disease progression, reduced viral RNA load	AG129	Zmurko et al., 2016
BCX4430	300mg/kg/day	Significantly improved survival when treatment was initiated during the peak of viremia	AG129	Julander et al., 2017
Ribavirin	500 mg/kg/day	Moderately reduced viremia, prolonged survival	STAT-1 KO	Kamiyama et al., 2017.
NITD008	50 mg/kg	Protected against lethal challenge, protected against neurological symptoms	A129	Deng et al., 2016
NSC157058	30 mg/kg/day	Reduced viral load in the blood	SJL	Shiryaev et al., 2017
Novobiocin	100 mg/kg 1dpi, 13dpi	Higher survival rate (100 vs. 0%), lower mean blood and tissue viral loads, and less severe histopathological changes	BALB/c mice	Yuan et al., 2017
Emetine	2 mg/kg/da	Reduced viral titer in blood plasma of SJL mice, reduced serum viral load in Ifnar1^−/−^, reduced NS1 protein and ZIKV RNA in serum and liver	SJL, A129	Yang et al., 2018
Cephaeline	2 mg/kg/day			
**INHIBITORS OF THE VIRUS-HOST CELL INTERACTIONS**				
Peptide Z2	10 mg/kg	Decreased viremia, blocked vertical transmission, protected against lethal challenge	A129, AG6	Li et al., 2017b
**INHIBITORS OF AUTOPHAGY AND MEMBRANE FUSION**				
Chloroquine	40 mg/kg^/^day; 50 mg/kg/day	Inhibited autophagy; decreased placental infection; attenuated mortality, blocked vertical transmission	A129, SJL	Cao et al., 2017; Shiryaev et al., 2017; Watanabe et al., 2017
25-Hydroxycholesterol	50 mg/kg	Reduced viremia, enhanced survival, inhibited infection and microcephaly in Fetal mice	A129	Li et al., 2017a

*A129 mice lack the receptor for IFN-α/β (type I interferon), STAT-1 KO have impaired type I interferon singling, AG129 mice lack receptors for IFN-α/β as well as receptors for IFN-γ (type II interferon), Swiss albino, BALB/C and SJL mice are interferon-competent*.

**Table 2:** Small-molecule inhibitors of ZIKV tested *in vitro*.

**Inhibitor**	**IC_**50**_ (μM)**	**ZIKV strain**	**Cell line**	**Primary assay**	**Secondary assay**	**Reference**
6-azauridine	2.30 ± 0.1	ZIKV^BR^; (KX197192.1)	Huh7	ZIKV ENV protein mAb 4G2 immunofluorescence (IF)	IFNα2A antibody IF	Pascoalino et al., 2016
5-fluorouracil	14.3 ± 8.6					
Palonosetron	16.3 ± 7.7					
Lovastatin	20.7 ± 8.6					
Kitasamycin	41.7 ± 10.1					
Sofosbuvir	1.37 ± 0.7*; 4.95 ± 0.86^∧^	PRVABC59	Huh7*, JAR^∧^	Plaque reduction assay	qRT-PCR from cell culture supernatants, ENV protein mAb 4G2 IF, *in vivo*	Bullard-Feibelman et al., 2017
	3.80 ± 0.67*; 2.10 ± 0.4^∧^	ZIKV^BR^				
	4.60 ± 0.82*; 3.79 ± 0.1^∧^	Dakar 41519				
	ND	ZIKV^BR^	Human fetal-derived hindbrain and cortex NSC	ENV ZV-64 mAb flow cytometry		
	1.90 ± 0.2*, 1.10 ± 0.2^#^, 0.41 ± 0.04^∧^, >50^@^	ZIKV^BR^	BHK-21*, SH-SY5Y^#^, Huh-7^∧^, Vero^@^, hNSC, brain organoids	Plaque reduction assay	Cell viability, caspase assay, ZIKV RdRp	Ferreira et al., 2017
Emetine	0.0298*	PRVABC59*, MR766^∧^, FSS13025	SNB-19*, HEK 293, Vero E6, hNSC	ENV mAB 4G2 IF, NS1 homogenous time-resolved fluorescence	Western blot, Plaque reduction assay, TDA, NS5 Polymerase inhibition, *in vivo* ZIKV suppression	Yang et al., 2018
Cephaeline	0.0189^∧^					
Brequinar	0.08*, 0.08^∧^	MR766^∧^, PRVABC59 ^∧^	Vero 76	Cytopathic effect inhibition (CPEi) via CellTiter-Glo	Titer-reduction, real-time PCR and Western blot	Adcock et al., 2017
NITD008	0.51*, 0.56^∧^					
CID 91632869	1.09*, 2.17^∧^					
Saliphenylhalamide	0.62*, 0.49^∧^				NA	
6-azauridine	3.18*, 3.91^∧^				titer-reduction, real-time PCR	
Mevastatin	3.42*, 5.05^∧^					
Finasteride	9.85*, 26.58^∧^					
Bromocriptine	13.04 ± 2.00	PRVABC59	Vero	CPEi via MTT, viral titer	Plaque reduction, NS2B-NS3 Protease assay	Chan et al., 2017
Bortezomib	~0.01	MEX_I_7	Huh-7	ENV mAb 4G2 IF	HeLa and JEG3 ENV assay, hNSC with flow cytometry	Barrows et al., 2016
NITD008	~0.50					
MPA	~0.80					
Daptomycin	~0.80					
Ivermectin	~5.00					
Chloroquine	~12.5–25.0*	MR766*, ZIKV^BR*^	Vero*, hBMECs, hNPC, mouse neurospheres	ENV mAb 4G2 flow cytometry and IF	Plaque reduction assay, Cell viability, qRT-PCR	Delvecchio et al., 2016
Curcumin	1.90	HD78788	HeLa	Plaque reduction assay	Cell viability, qRT-PCR, viral surface binding	Mounce et al., 2017
EGCG	21.4*	ZIKV^BR*^, MR766	Vero E6	Plaque reduction assay	Cell viability	Carneiro et al., 2016
GSK126	~10.0	ZIKV (H/PF/2013)	Human foreskin fibroblasts	Plaque forming assay	Pan-Flavivirus MAb E60	Arbuckle et al., 2017
Heparin	ND	MR766, INMI-1 (KU991811)	Human neural progenitor cells, Vero	ENV mAb 4G2 IF, CPEi via adenylate kinase assay	Caspase-3 IF, viral titer, cell viability	Ghezzi et al., 2017
Nanchangamycin	0.1*, 0.4^∧^, 0.97^#^	Mex2-81*^∧#^, MR766	U2OS*, HMBEC^∧^, Jeg-3^#^, EVTs, mouse primary midbrain neuron-glia mixed cultures	ENV mAb 4G2 IF	Cell viability, qRT-PCR, viral titer, TDA, viral uptake, transferrin	Rausch et al., 2017
Obatoclax	0.04 ± 0.01*	FB-GWUH-2016*, MR766, H/PF/2013, MRS_OPY_Martinique_PaRi_2015	mouse retinal pigment epithelium (RPE) cells	CPEi via CellTiter-Glo	TDA, drug combinations, qRT-PCR, plaque assays, cytokine profiling, IF, caspase-3/7/1 assays	Kuivanen et al., 2017
Saliphenylhalamide	0.5 ± 0.2*					
Gemcitabine	0.01 ± 0.0*					
Pentagalloylglucose	4.10	PRVABC59	Vero B4	qRT-PCR on culture supernatant	NA	Behrendt et al., 2017
Novobiocin	42.63*, 62.24^∧^	PRVABC59	Vero*, Huh-7^∧^	*in silico* NS2B-NS3 binding affinity, NS2b-NS3 protease activity	Fluorescence protease inhibition, *in vivo* validation	Yuan et al., 2017
Lopinavir-ritonavir	4.78 ± 0.41 μg/ml*, 3.31 ± 0.36 μg/ml^∧^					
Cn-716	0.25 ± 0.02	ZIKV^BR^	Crystallized NS2B-NS3^pro^	X-ray crystallography	CellTiter-Glo cytotoxicity assay for cn-716	Lei et al., 2016
Aprotinin	0.361 ± 19	ZIKV	Crystallized NS2B-NS3^pro^	X-ray crystallography	NA	Chen et al., 2016
Mefloquine	3.60	ZIKV^BR^	Vero	qRT-PCR on culture supernatant	Cell viability via XTT assay	Barbosa-Lima et al., 2017
Mefloquine analog 3a	0.80 ± 0.06					
Mefloquine analog 4	0.80 ± 0.03					
Merimepodib	0.60 ± 0.2*	FSS13025*, MR766	Huh-7, Vero E6	qRT-PCR on culture supernatant	Plaque reduction assay, CPEi via CellTiter-Glo	Tong et al., 2017
Various polyphenolics such as bis-indole alkaloids flinderole A* and flinderole B*, cassiarin D^∧^, cimiphenol^#^, 2′,4,4′-trihydroxy-3,3′-diprenylchalcone^@^	ND	Homology modeling of ZIKV proteins	NA	*in silico* molecular docking on NS2B/NS3 protease*, NS3 helicase^∧^,NS5 MTase^#^, NS5 RdRp^@^	NA	Byler et al., 2016
6-methylmercaptopurine riboside (6MMPr)	24.5*, 20.3^∧^	PE243 (KX197192.1)	Vero*, SH-SY5Y^∧^	qRT-PCR on culture supernatant	ZIKV ENV protein mAb 4G2 flow cytometry and IF, Plaque reduction assay	de Carvalho et al., 2017
Nordihydroguaiaretic Acid (NDGA^)^	9.10	ZIKV PA259459	Vero (CCL-81)	Plaque reduction assay	NA	Merino-Ramos et al., 2017
M4N	5.70					
2′-CMA	5.26 ± 0.12	MR766	Vero (CCL-81)	Plaque reduction assay	Flavivirus-specific antibody IF	Eyer et al., 2016
7-deaza-2′-CMA	8.92 ± 3.32					
2′-CMC	10.51 ± 0.02					
2′-CMG	22.25 ± 0.03					
2′-CMU	5.45 ± 0.64					
PKI 14-22	17.8	IbH 30656	HUVEC, astrocytes	Plaque reduction assay,	qRT-PCR, Western blot, TDA, RNA polymerase assay	Cheng et al., 2018
	22.3	MR766				
	34.1	H/FP/2013				
	19.2	PRVABC5				
GW4869	ND	MR766, PRVABC59	Human fetal astrocytes, Vero	qRT-PCR on culture supernatant	ENV mAb 4G2 IF, viral plaque assay, TDA	Huang et al., 2018
R848	Effective down to 0.1*	MR766	CHME3*, Primary human MDMs	intracellular qRT-PCR	Viperin Western blot	Vanwalscappel et al., 2018
CLR01	8.20*	MR766*	Vero E6*, HeLa, SW480, HFF, A172, H4, mouse cerebellum primary culture	CPEi via MTT*	ENV mAb 4G2 IF^∧#^, flow cytometry, TDA, qRT-PCR	Röcker et al., 2018
	6.7^∧^	FB-GWUH-2016^∧^				
	4.2^#^	PRVABC59^#^				

*Compounds with high selectivity and potency in each respective study*.

*TDA, time-of-drug addition assay; IF, immunofluorescence; CPEi, cytopathic effect inhibition assay; RdRP, RNA-dependent RNA polymerase*.

Superscript symbols *, ^∧^, ^#^, ^@^ designate corresponding samples/assays/cells/IC_50._

### Inhibitors of NS5 RNA Polymerase

Sofosbuvir, an FDA-approved inhibitor of the Hepatitis C virus RNA-dependent RNA polymerase (RdRp), is an effective inhibitor of ZIKV infection *in vitro*. Sofosbuvir blocks ZIKV replication by inhibiting the ZIKV RdRp and efficiently protects NPCs and three-dimensional neurospheres *in vitro* (Bullard-Feibelman et al., 2017; Ferreira et al., 2017). *In vivo*, post-infection treatment by sofosbuvir reduced acute levels of ZIKV by 90% in the blood plasma, brain, kidney and spleen, and doubled the percentage and survival time of immunodeficient mouse models (Ferreira et al., 2017; Mesci et al., 2018). Sofosbuvir also prevented acute neuromotor impairment and a loss of hippocampal- and amygdala-dependent memory. Most importantly, as mother-to-child ZIKV transmission results in microcephaly, sofosbuvir prevented the vertical transmission of ZIKV in pregnant mice. As a class B drug that has not been found to pose a risk to a developing fetus, and with acute treatment, it could in theory be used in pregnant women where the benefit in protecting against ZIKV could outweigh the risks to the unborn child.

ZIKV NS5 polymerase exhibits a high affinity interaction with the host cell importin a/b1 heterodimer, and these interactions can be blocked by the novel NS5-targeting inhibitor, N-(4-hydroxyphenyl) retinamide. This inhibitor has a potent anti-ZIKV activity at the low μM concentration and an established safety profile in humans. As a result, N-(4-hydroxyphenyl) retinamide holds promise as an anti-ZIKV agent (Wang et al., 2017b). ZIKV NS5 polymerase inhibitors (analogs of adenosine) 7-deaza-2'-C-methyladenosine and NITD008 that block the viral polymerase functions, also demonstrated promising results *in vitro* and *in vivo*, reducing viremia and delaying virus-induced morbidity and mortality in a ZIKV-infected AG129 mouse model (Deng et al., 2016; Zmurko et al., 2016).

Emetine, an antiprotozoal alkaloid derived from the ancient ipecac root, has recently been shown to demonstrate low nanomolar potency *in vitro* (IC_50_ = 30 nM) and *in vivo* against ZIKV and Ebola virus (Yang et al., 2018). Emetine was effective at 2 mg/kg/day in two different mouse models. Importantly, the drug also exhibited a good selectivity with IC_50_ values several-fold below the CC_50_ values. The work also revealed its dimethyl analog cephaeline had similar potency. The authors demonstrate two potential mechanisms of action including NS5 polymerase inhibition and modulation of host cell lysosomal function. Emetine is an FDA approved compound and could be rapidly translated from the bench to the field as a small molecule inhibitor of ZIKV replication. While emetine is contraindicated during pregnancy for the high doses required to inhibit amoebiasis, a larger safety margin may exist at the low doses required. Further work must be done to resolve whether emetine or its analog cephaeline are effective in preventing vertical transmission.

Ribavirin is a prodrug that resembles purine RNA nucleotides post-metabolism. It has been found to interfere with RNA processing required for viral replication. Ribavarin, historically used to treat hepatitis C in combination with interferon (IFN), now replaced by direct-acting antivirals, has been shown to inhibit ZIKV replication and ZIKV-induced cell death without cytotoxic effects (Kamiyama et al., 2017). Furthermore, ribavirin abrogated viremia in ZIKV-infected STAT-1-deficient mice, which succumb to ZIKV as STAT-1 is essential for type 1 IFN signaling (Kamiyama et al., 2017). However, ribavirin is classified as Category X and is strictly contraindicated during pregnancy or in those who are planning to become pregnant within 6 months of taking the drug.

The adenosine analog BCX4430 with broad-spectrum activity against a wide range of RNA viruses, including yellow fever, Marburg and Ebola viruses inhibited ZIKV cytopathic effects *in vitro* and in AG129 mice (Julander et al., 2017). Treatment of ZIKV-infected mice with BCX4430 significantly improved outcome even when treatment was initiated during the peak of viremia (Julander et al., 2017). As with many of these anti-ZIKV agents, it is vital to demonstrate the utility of BCX4430 in preventing congenital infection and sexual transmission. Surprisingly, in contrast to studies in STAT-1deficient mice (Kamiyama et al., 2017), ribavirin did not improve the outcome of ZIKV infection in AG129 mice in this study (Julander et al., 2017).

### Inhibitors of NS2B-NS3 Protease

In addition to the inhibitors of ZIKV NS5 polymerase, the inhibitors of the viral NS2B-NS3 protease demonstrated a good potential for the therapeutic development. Taking advantage of the previously discovered allosteric inhibitors of the West Nile virus NS3 proteinase (Shiryaev et al., 2011), researchers identified structural scaffolds for allosteric small-molecule inhibitors of ZIKV NS2B-NS3 protease (Shiryaev et al., 2017). Molecular modeling of the protease-inhibitor complexes suggested that NSC157058 binds to the druggable cavity in the NS2B-NS3 protease interface and affects productive interactions of the protease domain with its cofactor. The most potent compound demonstrated efficient inhibition of ZIKV propagation in human fetal NPCs and in SJL mice (Shiryaev et al., 2017).

By screening over 2,800 approved and investigational drugs, Li et al. (2017d) identified temoporfin, nitazoxanide, and niclosamide as inhibitors of the ZIKV protease. These three compounds blocked the productive interactions of the viral NS2B co-factor with the NS3 protease domain and inhibited the NS2B-NS3 protease activity with nanomolar potency. Temoporfin, a clinically approved drug, demonstrated the most pronounced effect by protecting NPCs and placental cells, and reducing viremia and mortality in mice. Interestingly, temoporfin also has potential as a pharmaceutical agent against other flaviviruses (Sacramento et al., 2017).

More recently, studies show that viperin (virus inhibitory protein, endoplasmic reticulum-associated, interferon-inducible, also known as RSAD2) restricted ZIKV and tick-borne encephalitis virus replication by targeting NS3 for proteasomal degradation (Panayiotou et al., 2018). Viperin was found to interact and co-localize with ZIKV NS2A, NS2B, and NS3. Interestingly, viperin expression reduced the NS3 protein level post-infection, and the stability of the other interacting viral proteins, but only in the presence of NS3. Although viperin interacted with NS3 from several mosquito-borne flaviviruses (ZIKV, Japanese encephalitis virus, and yellow fever virus), only ZIKV was sensitive to the antiviral effect of viperin (Panayiotou et al., 2018). However, the *in vivo* efficacy of viperin has not yet been investigated.

### Host Targets of Anti-ZIKV Drugs

An additional therapeutic approach that could be implemented against ZIKV is the blockade of the virus-host cell interactions. For example, a synthetic peptide Z2, that represents a portion of the ZIKV envelope protein, was able to efficiently inhibit ZIKV infection by interacting with ZIKV surface proteins and disrupting the viral membrane integrity (Li et al., 2017b). Peptide Z2 inhibited ZIKV infection not only *in vitro*, but also *in vivo*, due its ability to penetrate the placental barrier and to prevent ZIKV vertical transmission in C57BL/6 pregnant mice.

Chloroquine, a common antimalarial agent, is a good example of the FDA-approved drugs that could be repurposed for the treatment and prophylaxis of ZIKV infection. Several independent laboratories demonstrated that chloroquine efficiently blocks ZIKV infection in human NPCs (Delvecchio et al., 2016) and limits vertical transmission *in vivo* (Cao et al., 2017; de Carvalho et al., 2017; Shiryaev et al., 2017). Chloroquine (and its derivative hydroxychloroquine) inhibits autophagy, which is the proposed mechanism of its anti-ZIKV action since deficiency in an essential autophagy gene, Atg16l1, in mice limited ZIKV vertical transmission, placental and fetal damage, and improved placental and fetal outcomes overall (Cao et al., 2017). Chloroquine extended the lifespan of ZIKV-infected INF signaling-deficient AG129 mice. Treatment of ZIKV-infected pregnant SJL mice during mid- to late-gestation reduced the levels of ZIKV in the fetal brain over 20-fold (Shiryaev et al., 2017). Because chloroquine has limited to no side-effects in pregnant women, and is available, and affordable worldwide, this pharmaceutical is a natural candidate for human trials for ZIKV treatment and prophylaxis.

A natural metabolite produced by immune cells in response to infection, 25-hydroxycholesterol (25HC) was demonstrated to block viral entry into host cells (Li et al., 2017a). As a response to ZIKV infection, cholesterol-25-hydroxylase is induced in the infected cells and its product, 25HC, efficiently protects against ZIKV infection as a natural antiviral compound. Synthetic 25HC inhibited ZIKV infection *in vitro*. In a mouse model of microcephaly, 25HC reduced viremia and tissue damage in adult mice, as well as brain damage in embryos. Similar results were observed with 25HC in rhesus macaques.

**Figure 2:**
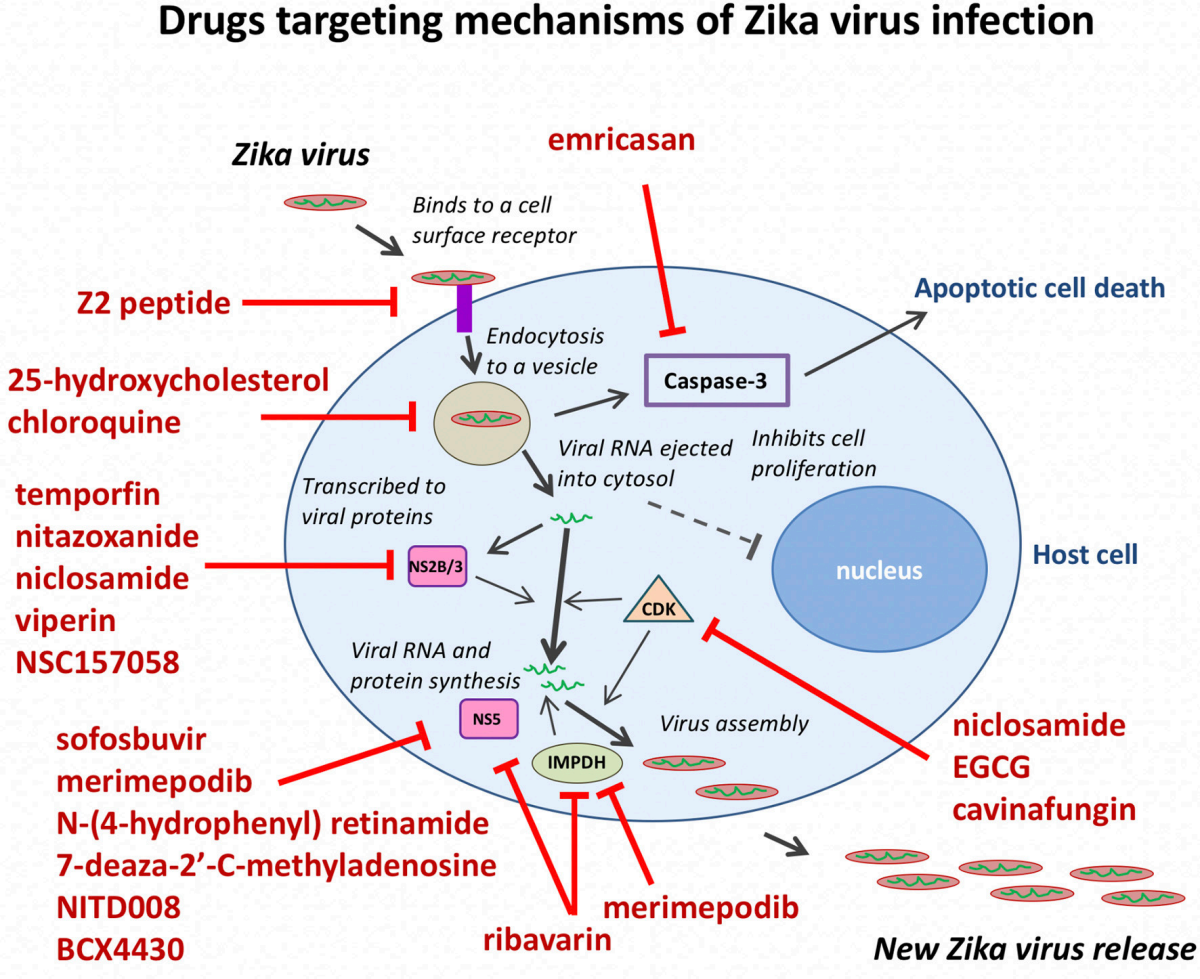
Anti-ZIKV drugs targeting the different steps in the viral life cycle. Emricasan targets caspase-3 activity to prevent cell death. Z2 peptide targets ZIKV cell entry. 25-hydroxycholesterol and chloroquine interfere with lipid homeostasis and autophagy to disrupt viral particle release after endocytosis. Temoporfin, nitazoxanide, niclosamide, and viperin block NS2B/NS3 protease activity to prevent viral replication. Sofosbuvir, merimepodib, N-(4-hydrophenyl) retinamide, 7-deaza-2′-C-methyladenosine, NITD008, BCX4430, and ribavarin block ZIKV NS5 polymerase activity to prevent viral replication. Ribavarin and merimepodib block IMPDH, an enzyme involved in *de novo* synthesis of guanine nucleotides. Niclosamide, EGCG, and cavinafungin block CDKs and prevent viral replication.

Merimepodib (VX-497) is a potent inhibitor of inosine-5′-monophosphate dehydrogenase (IMPDH), an enzyme involved in *de novo* synthesis of guanine nucleotides, and exhibits antiviral activity against HCV and a variety of DNA and RNA viruses *in vitro*. Tong et al. (2017) expanded the antiviral spectrum of Merimepodib and demonstrated its ability to inhibit ZIKV RNA replication (IC_50_ = 0.6 μM) and to reduce the production of ZIKV in Vero cells. Merimepodib, especially if used in combination with other antivirals such as ribavirin and T-705 (favipiravir), can enhance suppression of virus production. The targets of many ZIKV inhibitors during the viral life cycle are illustrated in Figure 2.

Taken together, these recent accomplishments provide a strong foundation for the development of safe, potent, and selective anti-ZIKV therapeutics. Because of its unique pathology, ZIKV is an elusive virus for any drug discovery program. However, an understanding of ZIKV biology and host-cell interactions greatly aid in developing therapeutic solutions. For pregnant women, it would be best to develop or find those drugs that would be classified as Category B or lower with minimal or no risk to the developing fetus and pregnant mother. All public health organizations must continue to be vigilant and maintain an in-depth understanding how ZIKV affects the individual and global health. Armed with the elucidated mechanisms of action of the multiple anti-ZIKV compounds described in this study, synergistic drug combinations could be developed that would target distinct steps of ZIKV infection. This approach is likely to generate a more effective therapy and prevent against the possibility of ZIKV drug resistance. As advancements in cell biology and cell culture techniques continue to provide new avenues for ZIKV drug studies, the more complex *in vitro* models such as three-dimensional brain organoids that readily mimic human brain tissue will provide a wealth of knowledge about virus-host interactions and produce viable drugs to prevent infection (Watanabe et al., 2017).

## Perspective and Outlook

ZIKV drug discovery efforts have culminated in a long list of potentially effective drug candidates as evidenced by the *in vivo* tested drugs listed in Table 1 and the *in vitro* tested drugs in Table 2. The proportion of compounds that will make it from the *in vitro* list to the *in vivo* list is yet unknown. Nonetheless, due to the common practices of drug discovery, lead hits must typically first demonstrate *in vitro* activity and, in this way, obtain value in order to warrant further *in vivo* exploration. From the compounds tested in mice, the proportion of drugs that make it to the clinic is even smaller. To date, there are only handful of ongoing clinical trials for ZIKV therapies. None of these so far include small molecules therapies, and most current trials are ZIKV vaccine candidates. For example, a safety and tolerability trial for a ZIKV monoclonal antibody called tyzivumab is enrolling patients at the SingHealth Investigational Medicine Unit (ClinicalTrials.gov Identifier: NCT03443830, Sponsor: Tychan Pte Ltd.). Another safety trial for a ZIKV purified inactivated vaccine (ZIVP) has been ongoing since November 2016 at St. Louis University Center for Vaccine Development (ClinicalTrials.gov Identifier: NCT03443830, Sponsor: NIAID). These 13 trials include safety, tolerability, and immunogenicity studies for inactivated ZIKV vaccines, a live attenuated vaccine, DNA vaccines, and an mRNA vaccine.

So far, only two of ZIKV clinical trials have been completed, and both are for ZIKV vaccine candidates: one for GLS-5700, a DNA plasmid vaccine encoding for the premembrane-membrane, and envelope regions of Zika virus, and another for a vaccine called MV-ZIKA. None of these trials thus far have included efficacy studies. These trials exemplify the global fight against ZIKV with study locations ranging from the U.S. to Singapore to Austria. NIAID is sponsoring a trial for a Zika virus wildtype DNA vaccine called VRC 705 (ClinicalTrials.gov Identifier: NCT03110770) with locations in the US, Brazil, Colombia, Costa Rica, Ecuador, Mexico, Panama, Peru, and Puerto Rico. These are all locations with previously known ZIKV activity and are at risk for future ZIKV outbreaks.

As is evident by the several trials described above, safety is a key characteristic of this effort as ZIKV has only mild effects in most adult patients. However, the danger for ZIKV lies in its ability to severely disrupt the development of newborn children by transferring from mother to child or from man to woman and vice versa during sexual intercourse furthering the risk to the general population. The effects of ZIKV have already produced a generation of children with microcephaly who will never lead a normal life. The field must push forward vaccine, biologic, and small molecule drug compounds through the pipeline more efficiently with a stronger focus on FDA-approved compounds to be accepted into clinical practice at a faster rate. Scientists and communities should press their governments to be vigilant in obtaining the funds necessary to do this much needed research. The threat of ZIKV is not over. Re-emergence of potentially catastrophic viral epidemics should evoke in all of us the need to push forward. After all, most people around the world have never been exposed to the virus and are therefore immunologically naive to combat a ZIKV infection. In sum, with the work described above, there is a promising future that we may 1 day be protected against ZIKV and the like, but we must not let down our guard.

## Author Contributions

AT and SF conceived the manuscript and wrote parts of the manuscript. SS, Y-WL, C-TH, AP, CF, AS, wrote parts the manuscript and edited the manuscript. KG and WZ wrote parts of the manuscript, edited the manuscript, and generated the figures.

### Conflict of Interest Statement

The authors declare that the research was conducted in the absence of any commercial or financial relationships that could be construed as a potential conflict of interest.
